# Establishment of a nomogram for predicting the surgical difficulty of anterior cervical spine surgery

**DOI:** 10.1186/s12893-020-01022-0

**Published:** 2021-03-29

**Authors:** Chengyue Ji, Yuluo Rong, Jiaxing Wang, Guoyong Yin, Jin Fan, Pengyu Tang, Dongdong Jiang, Wei Liu, Xuhui Ge, Shunzhi Yu, Weihua Cai

**Affiliations:** 1 Department of Orthopedics, The First Affiliated Hospital of Nanjing Medical University, 300 Guangzhou Road, Nanjing, Jiangsu China; 2 Department of Orthopedics, Shanghai Tenth People’s Hospital, Tongji University School of Medicine, 301 Yanchang Road, Shanghai, China

**Keywords:** Surgical difficulty, Anterior cervical spine surgery, Nomogram

## Abstract

**Background:**

For a long time, surgical difficulty is mainly evaluated based on subjective perception rather than objective indexes. Moreover, the lack of systematic research regarding the evaluation of surgical difficulty potentially has a negative effect in this field. This study was aimed to evaluate the risk factors for the surgical difficulty of anterior cervical spine surgery (ACSS).

**Methods:**

This was a retrospective cohort study totaling 291 consecutive patients underwent ACSS from 2012.3 to 2017.8. The surgical difficulty of ACSS was defined by operation time longer than 120 min or intraoperative blood loss equal to or greater than 200 ml. Evaluation of risk factors was performed by analyzing the patient’s medical records and radiological parameters such as age, sex, BMI, number of operation levels, high signal intensity of spinal cord on T2-weighted images, ossified posterior longitudinal ligament (OPLL), sagittal and coronal cervical circumference, cervical length, spinal canal occupational ratio, coagulation function index and platelet count.

**Results:**

Significant differences were reported between low-difficulty and high-difficulty ACSS groups in terms of age (*p* = 0.017), sex (*p* = 0.006), number of operation levels (*p* < 0.001), high signal intensity (*p* < 0.001), OPLL (*p* < 0.001) and spinal canal occupational ratio (*p* < 0.001). Multivariate logistic regression analysis revealed that number of operation levels (OR = 5.224, 95%CI = 2.125–12.843, p < 0.001), high signal intensity of spinal cord (OR = 4.994, 95%CI = 1.636–15.245, p = 0.005), OPLL (OR = 6.358, 95%CI = 1.932–20.931, p = 0.002) and the spinal canal occupational ratio > 0.45 (OR = 3.988, 95%CI = 1.343–11.840, p = 0.013) were independently associated with surgical difficulty in ACSS. A nomogram was established and ROC curve gave a 0.906 C-index. There was a good calibration curve for difficulty estimation.

**Conclusion:**

This study indicated that the operational level, OPLL, high signal intensity of spinal cord, and spinal canal occupational ratio were independently associated with surgical difficulty and a predictive nomogram can be established using the identified risk factors. Optimal performance was achieved for predicting surgical difficulty of ACSS based on preoperative factors.

## Background

Anterior cervical spine surgery (ACSS) is a well-established surgical intervention that has demonstrated long-term clinical benefits in a number of spine conditions such as in the management of degenerative cervical spine disease. However, despite its wide application, ACSS is associated with complicated and difficult surgical processes which are often accompanied by high risk and cost. Some of the potential risks that can be disastrous include esophageal injury, epidural hematoma, airway obstruction and vascular injury [1–5]. The surgical difficulty is a pressing concern among surgeons and patients since it highly correlates with the safety and effectiveness of the surgical process. Previously, the evaluation of surgical difficulty has been based on subjective perception rather than objective indexes. In addition, there is a lack of systematic research with regard to the evaluation of surgical difficulty in ACSS. The scientific multi-criteria system is important in decision-making for a surgical process, physician–patient communication, and surgical skills training. This study aimed to evaluate the risk factors for surgical difficulty of anterior cervical spine surgery (ACSS).

## Methods

### Study population

A retrospectively maintained surgical database of 356 consecutive patients who underwent anterior cervical surgery in our center from March 2012 to August 2017 was reviewed. All the patients received written informed consent prior to the surgery. The study was approved by our local institutional ethics committee. The indications for anterior surgery were cervical myelopathy, radiculopathy and ossified posterior longitudinal ligament (OPLL). The exclusion criteria were trauma, tumor, infection, previous cervical surgery and lack of clear preoperative imaging data. Therefore, we analyzed clinical data of 291 patients who underwent anterior cervical spine surgery.

### Surgical procedure

All patients were performed by an experienced spine surgeon (C.W.). The choice of operation was primarily dependent on spinal cord compression. Corpectomy has been effectively used to treat various pathologies including osteophytes, OPLL and nucleus pulposus extending posteriorly to the vertebral body. In this study, discectomy was used if the compression was limited to the disk level. Anterior cervical discectomy and corpectomy were performed with the Smith–Robinson technique. In corpectomy, the optimal titanium mesh cage (DePuy Spine, New Brunswick, New Jersey) was filled with morselized autologous bone and positioned into the defect. In discectomy, PEEK interbody cage (DePuy Spine, New Brunswick, New Jersey) was inserted into the disk space. Anterior cervical locking plates with variable angle screws (DePuy Spine, New Brunswick, New Jersey) were employed in all the patients. Patients were instructed to wear a cervical collar for 6 weeks postoperatively.

### Definitions

**Fig. 1:**
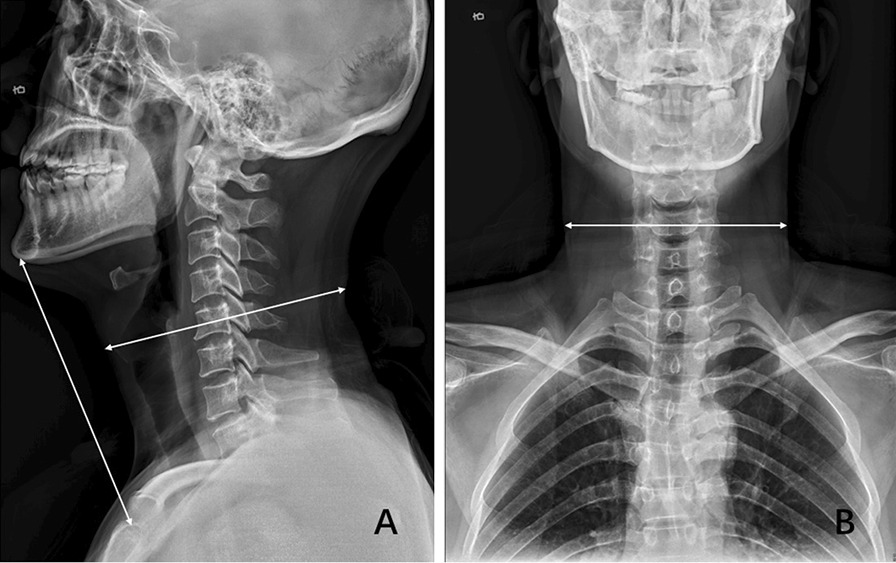
Sagittal radiograph showing that the cervical length was measured as the distance between the sternum and gnathion. Sagittal and coronal cervical circumferences were defined as the distance between the intersection of the line parallel to the endplate through the midpoint of C5 and soft tissue shadow

**Fig. 2:**
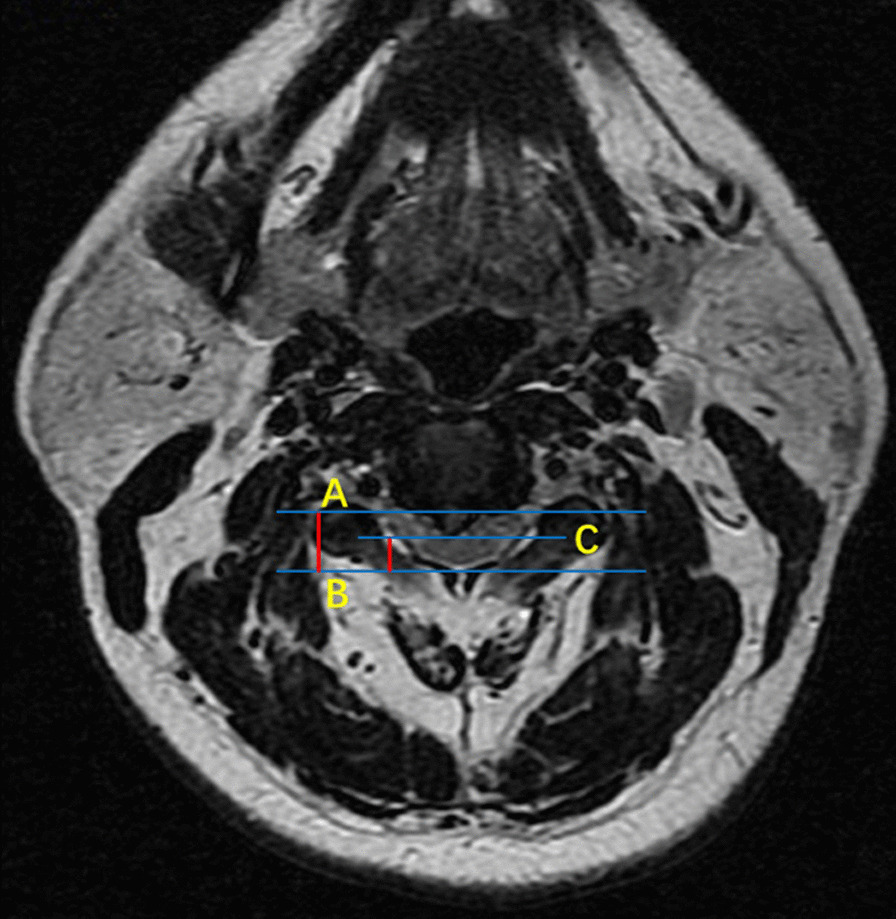
At the most compressed level, the anteroposterior diameter was defined as the distance between the posterior edge of vertebral body and spinal canal (**a**, **b**). The shortest diameter was the distance between the posterior edge of compression and spinal canal (**b**, **c**). Spinal canal occupational ratio = (anteroposterior diameter-shortest diameter) / anteroposterior diameter

The mean time of surgery in this study was 120.3 min which was chosen as the cut-off. Operation time longer than 120 min or intraoperative blood loss equal to or greater than 200 ml was considered as a high-difficult surgery. Otherwise, it was defined as low-difficulty surgery. Examined radiological parameters included high signal intensity of spinal cord on T2-weighted images, OPLL, sagittal cervical circumference, coronal cervical circumference, cervical length, anteroposterior diameter of the spinal canal, the shortest diameter of the spinal canal, and spinal canal occupational ratio. Sagittal and coronal cervical circumferences were defined as the distance between the intersection of the line parallel to the endplate through the midpoint of C5 and soft tissue shadow (Fig. 1). Cervical length was measured as the distance between the sternum and gnathion on the lateral radiograph (Fig. 1a). The anteroposterior and shortest diameter of the spinal canal were measured at the most compressed level using standard picture archiving and communication system (PACS). Anteroposterior diameter was the distance between the posterior edge of the vertebral body and spinal canal while the shortest diameter was defined as the distance between the posterior edge of the compression and spinal canal. The following formula was used to calculate the spinal canal occupational ratio: spinal canal occupational ratio = (anteroposterior diameter-shortest diameter)/anteroposterior diameter (Fig. 2).

### Statistical analysis

SPSS, version 23.0 and R, version 3.6.1 were used to perform all the data analysis. Continuous variables were expressed as mean ± standard deviation. Comparisons between the high-difficulty group and low-difficulty group were performed using Student’s t-test, Mann–Whitney U test, χ^2^ test. Potential risk factors were identified by multivariate logistic regression analysis (p values < 0.05). The predictive value of the multivariate logistic regression was visualized by the receiver operating characteristics (ROC) curves. A nomogram was established using the rms package of R, version 3.6.1. The concordance index (C index) and a calibration curve were used to determine predictive accuracy. A *P* value of less than 0.05 was considered statistically significant.

## Results

### Patient demographics and clinical characteristics

Detailed participants’ demographics and clinical characteristics in our study are given in Table1. All patients were divided into high-difficulty group (n = 140, 48.1%) and low-difficulty group (n = 151, 51.9%). Among the 291 patients, 180 (61.8%) were male while 111 (38.2%) were female, with a mean age of 52.3 (range 18–76). The mean surgical time was 120.3 min (60–250 min) and mean blood loss was 33.1 ml (5–400 ml). In the low-difficulty group, one patient had cerebrospinal fluid leakage and two patients had dysphagia. Whereas in the high-difficulty group, the main complications reported were cerebrospinal fluid leakage in three patients, epidural hematoma in one patient and dysphagia in four patients.

**Table 1:** Comparison of the low-difficulty and high-difficulty groups

Factor	Low-difficulty group (N = 151)	High-difficulty group (N = 140)	*P*
Age*(years)	50.99 ± 10.60	53.84 ± 9.62	0.017
Sex* (male: female)	82:69	98:42	0.006
BMI	24.15 ± 3.07	24.74 ± 2.82	0.154
Number of operation levels*			
1	86	7	
2	56	77	
3	9	53	
4	0	3	< 0.001
High signal intensity of spinal cord*			
Yes	52	96	< 0.001
No	94	49	
OPLL*			
Yes	17	74	< 0.001
No	136	64	
Sagittal Cervical Circumference	14.36 ± 1.99	14.86 ± 2.14	0.053
Coronal Cervical Circumference	12.41 ± 1.41	12.74 ± 1.38	0.057
Cervical Length	14.56 ± 2.10	14.63 ± 1.83	0.819
Occupying Ratio*	0.37 ± 0.15	0.49 ± 0.11	< 0.001
PT	11.70 ± 0.66	11.74 ± 0.71	0.708
INR	1.01 ± 0.05	1.02 ± 0.06	0.660
APTT	28.06 ± 2.50	27.90 ± 2.71	0.684
FIB	2.64 ± 0.98	2.44 ± 0.73	0.130
TT	18.26 ± 1.03	18.50 ± 1.46	0.189
DD2	0.32 ± 0.37	0.33 ± 0.59	0.924
PLT	213.82 ± 79.45	201.49 ± 51.96	0.210

*BMI* body mass index, *OPLL* ossification of the posterior longitudinal ligament

*Statistically significant difference

### Establishment of a nomogram for predicting surgical difficulty

**Fig. 3:**
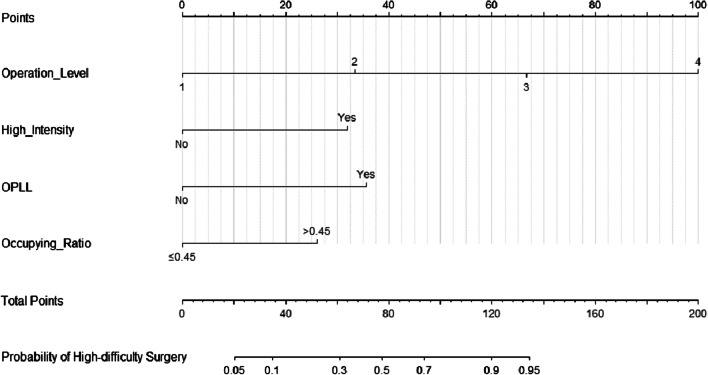
A predictive nomogram was established by combining the four risk factors

Comparisons between the low-difficulty and high difficulty groups showed that age (*p* = 0.017), sex (*p* = 0.006), number of operation levels (*p* < 0.001), high signal intensity (*p* < 0.001), OPLL (*p* < 0.001) and spinal canal occupational ratio (*p* < 0.001) significantly correlated with surgical difficulty. The ROC curves further showed that the best thresholds for age and spinal canal occupational ratio were 55 (sensitivity: 53.2%; specificity: 68.9%) and 0.45 (sensitivity: 74.2%; specificity: 70.5%). Multivariate logistic regression analysis revealed that number of operation levels (OR = 5.224, 95%CI = 2.125–12.843, p < 0.001), high signal intensity (OR = 4.994, 95%CI = 1.636–15.245, p = 0.005), OPLL (OR = 6.358, 95%CI = 1.932–20.931, p = 0.002) and the spinal canal occupational ratio > 0.45 (OR = 3.988, 95%CI = 1.343–11.840, p = 0.013) were independent risk factors for surgical difficulty of anterior cervical surgery (Table 2). Based on these results, number of operation levels, high signal intensity of spinal cord, OPLL and spinal canal occupational ratio can be used to establish a nomogram for use in predicting surgical difficulty (Fig. 3).

**Table 2:** Multivariate logistic regression analysis

Factor	Odds ratio (95% CI)	*P*
Age > 55	1.507 (0.505–4.494)	0.462
Sex	0.708 (0.224–2.241)	0.557
Number of operation levels	5.224 (2.125–12.843)	< 0.001
High signal intensity	4.994 (1.636–15.245)	0.005
OPLL	6.358 (1.932–20.931)	0.002
Occupying Ratio > 0.45	3.988 (1.343–11.840)	0.013

CI indicates confidence interval

*OPLL* ossification of the posterior longitudinal ligament

### Evaluation of prediction nomogram

**Fig. 4:**
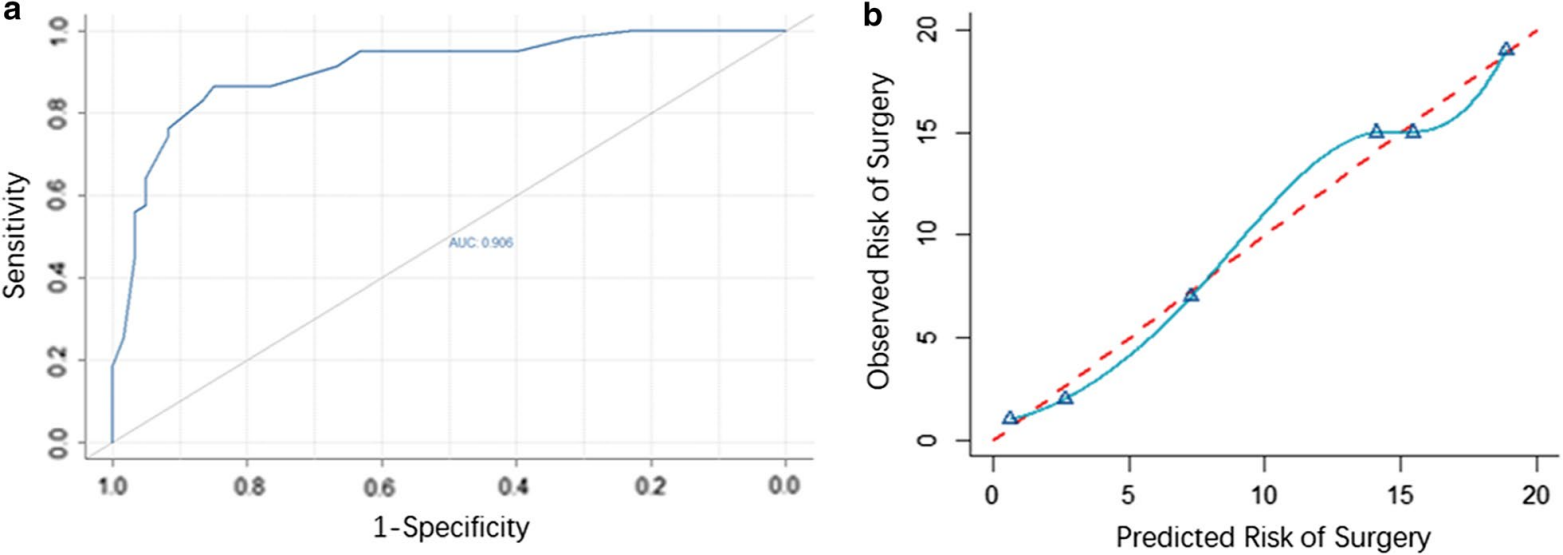
The ROC curve (**a**) demonstrated that the C-index was 0.906. **b** The calibration curve showed that the probability of high-difficulty predicted by the nomogram agreed well with actual practice

The ROC curve for the nomogram model showed that the C-index was 0.906, which was an indication of a robust discriminative ability (Fig. 4a). The calibration curve demonstrated that the probability of high-difficulty predicted by the nomogram model agreed well with actual practice (Fig. 4b).

## Discussion

As the population ages, the incidence of degenerative cervical disease is continuously increasing, which is the foremost cause of neurological deficit among adults worldwide [6, 7]. There is a paradigm shift in the treatment of degenerative cervical disease from posterior to anterior decompression, due to the direct removal of spinal cord compressions, such as disc herniation, osteophytes, and OPLL [8–10]. However, the anterior approach is associated with potential risks that can cause life-threatening complications in vulnerable organs anterior to the vertebral body, such as trachea, esophagus and blood vessels [3]. Therefore, cervical spine surgery is associated with higher surgical difficulty than lumbar surgery hence the need to evaluate surgical difficulty and the associated risk factors for optimal performance in ACSS. From the perspective of developing surgical protocols, assessment of surgical procedure and surgical skill training, a classification system determining the difficulty level is extremely important. Moreover, adequate physician–patient communication is necessary for patients with high surgical difficulty.

Researchers have established several scoring systems for assessing the surgical difficulty. Natkaniec et al. [11] demonstrated that gender, tumor size, and localization are some of the parameters associated with the level of the surgical difficulty of the laparoscopic lateral transperitoneal adrenalectomy. Hasegawa et al. [12] established a novel model for the prediction of surgical difficulty in laparoscopic liver resection and included factors such as the extension of resection, location of tumor, obesity and platelet count. However, a preoperative scoring system evaluating the surgical difficulty of ACSS has not been reported. Therefore, due to lack of a gold standard to estimate for surgical difficulty, surrogate indicators are often used to indirectly reflect the difficulty and these include operative time, intraoperative blood loss and incidence of complication [11–14]. High-difficulty ACSS has been correlated with longer operative time and more intraoperative bleeding.

In this study, a number of parameters such as age, sex, BMI, number of operation levels, high signal intensity of spinal cord on T2-weighted images, OPLL, sagittal cervical circumference, coronal cervical circumference, cervical length, spinal canal occupational ratio, and coagulation function index were evaluated to identify risk factors for high-difficulty ACSS. Previous studies confirm that the elderly, obese patients and male gender are associated with longer operative time [11, 12, 15]. However, this study reported that there is no significant difference in terms of age, sex, and BMI between low-difficulty and high-difficulty groups of ACSS. Coagulation function index and platelet count are associated with intraoperative hemorrhage, however, no statistical significance in these factors was reported in our study. To our knowledge, thickness of neck might influence the operative time due to the difficulty of surgical exposure. Although surgical time tend to be longer in patients with thick neck, this trend did not reach statistical significance in our study.

Operational level was a vital factor affecting the difficulty of ACSS. In our study, as operational segments increased, prolonged operative time and more intraoperative blood loss were reported and these findings were consistent with previous research [16]. Moreover, the incidence of complications associated with the surgical procedure increased when more segments were decompressed and fused. Kou et al. [17] reported that multilevel procedures had a significantly higher risk for epidural hematoma. Sagi et al. [1] also reported that the number of vertebral bodies exposed more than three levels and operative time longer than 5 h were statistically associated with increased risk for airway complications with potentially catastrophic consequences. The selection of operational levels is mainly based on detailed clinical evaluation including extent of compression, chief complaint and the patient’s general condition. Adequate neurological decompression is essential for optimal clinical outcomes but caution should be made to reduce the affected segments to minimize surgical trauma and risk of potential complications.

The study demonstrated that OPLL was another predictive factor for high-difficulty surgery. The technical challenges faced when treating OPLL depend on the decompression procedure including less injury to the spinal cord and better protection of the venous plexus especially when combining dural ossification [18]. Furthermore, OPLL is associated with high risks of perioperative complications, such as cerebrospinal fluid leakage, dysphonia, dysphagia and neurological impairment [19]. Spinal canal occupational ratio was used to represent the degree of compression and high signal intensity of spinal cord on T2-weighted images closely correlated with severe spinal cord compression [20]. This study confirmed that the spinal canal occupational ratio and high signal intensity were risk factors for surgical difficulty. Therefore, surgeons should be cautious when severe compression and high signal intensity are observed on MR images.

For the clinical use of the established nomogram, a 0.906 C-index was reported for the model in addition to a good calibration curve for use in predicting the probability of high-difficulty surgery.

There are several limitations of this study that need to be addressed. First, this is a retrospective study. Second, this study was conducted in a single institution and the limited number of cases might restrict the value of its clinical use. Due to these limitations, prospective multi-center studies with a large sample size are needed to validate our results.

## Conclusions

This study indicated that the number of operational levels, OPLL, high signal intensity of spinal cord, and spinal canal occupational ratio were independently associated with surgical difficulty and a predictive nomogram can be established using the identified risk factors. Optimal performance was achieved for predicting surgical difficulty of ACSS based on preoperative factors.

## Data Availability

The data are not publicly available due the privacy of patients included but are available from the corresponding author on reasonable request for academic research purpose.

## Abbreviations

ACSS: Anterior cervical spine surgery
OPLL: Ossified posterior longitudinal ligament
PACS: Picture archiving and communication system
ROC: Receiver operating characteristics (ROC) curve
OR: Odds ratio
